# An Innocent Esophageal Mucosal Bridge: Case Report and Literature Review

**DOI:** 10.1177/2324709618767204

**Published:** 2018-03-28

**Authors:** Tagore Sunkara, Eric Omar Then, Krishna Sowjanya Yarlagadda, Manan Jhaveri, Vinaya Gaduputi

**Affiliations:** 1 The Brooklyn Hospital Center, Clinical Affiliate of The Mount Sinai Hospital, Brooklyn, NY, USA; 2 SBH Health System, Bronx, NY, USA; 3 Southwest Community Health Center, Bridgeport, CT, USA

**Keywords:** mucosal bridge, dysphagia, esophagus, endoscopy

## Abstract

An esophageal mucosal bridge is a rare finding that is seldom encountered on upper endoscopy. They most commonly present secondary to an underlying inflammatory disorder and cause chest pain and dysphagia, among other symptoms. More rarely, they present asymptomatically with no identifiable inflammatory conditions. Our case consists of a 31-year-old woman who presented with an asymptomatic, noninflammatory mucosal bridge of the esophagus. To our knowledge, this makes the third such case. The rarity of this condition coupled with the lack of epidemiologic data available make this case worthy for literature review.

## Introduction

Mucosal bridges are linear extensions of smooth muscle that connect across the lumen of the gastrointestinal tract. The resulting connection gives the appearance of a “double lumen” or “pseudo-carina” when visualized on upper endoscopy.^1^ Cases of mucosal bridges are rare, with reported sites including the esophagus, gastric antrum, small intestine, colon, and biliary tree.^2^ Clinically, symptoms vary greatly depending on their location, and are at times asymptomatic. Of note, there have been reported cases of mucosal bridges presenting in the vocal cords but are outside the scope of this discussion.^3^

## Case Report

**Figure 1.**
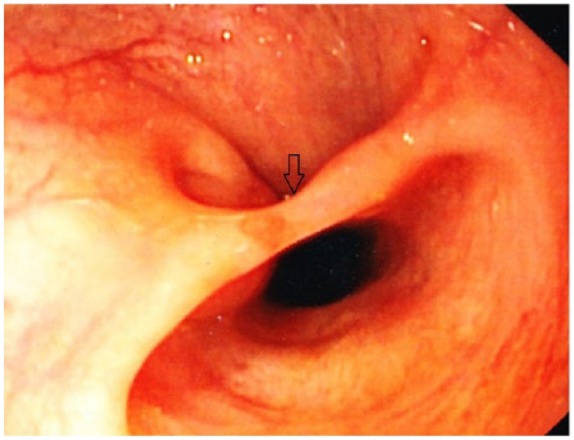
Esophagogastroduodenoscopy revealed a mucosal bridge (black arrow) across the lower third of the esophagus on view 1.

**Figure 2.**
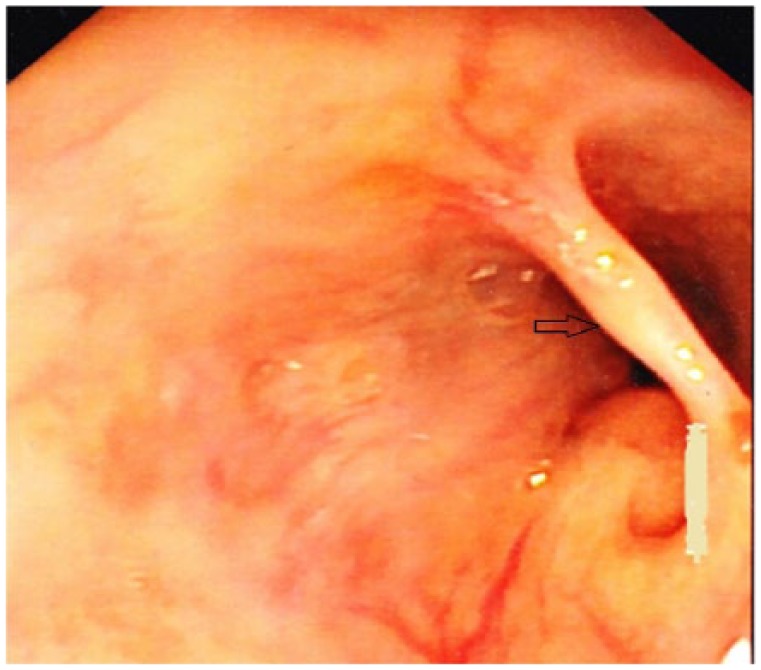
Esophagogastroduodenoscopy revealed a mucosal bridge (black arrow) across the lower third of the esophagus on view 2.

A 31-year-old woman with medical comorbidities of HIV on antiretroviral therapy and granulomatous hepatitis related to *Mycobacterium avium intracellulare* presented to the emergency department complaining of fatigue and malaise of 5 days duration. The patient denies any fevers, headache, nausea, vomiting, abdominal pain, diarrhea, constipation, bright red blood per rectum, or heavy menstruation. She claimed she is compliant with her medications. On presentation, the patient was afebrile with blood pressure of 138/78 mm Hg, heart rate of 84 beats per minute, and 98% saturation on room air. Physical examination revealed pallor but otherwise not significant. Laboratory workup revealed severe anemia with hemoglobin of 6 g/dL and mean corpuscular volume of 78 fL. Iron profile revealed iron levels of 40 µg/dL and total iron binding capacity of 440 µg/dL; otherwise blood work was not significant. The patient was transfused 2 units of packed red blood cells. As a part of the anemia workup, a diagnostic endoscopy and colonoscopy was scheduled. Esophagogastroduodenoscopy revealed a mucosal bridge across the lower third of the esophagus giving the appearance of “pseudo-carina” (Figures 1 and 2). Endoscopic appearance did not suggest any underlying active inflammatory mucosal pathology. As the patient was asymptomatic and had not complained of dysphagia or odynophagia, the mucosal bridge was not manipulated. Esophagogastroduodenoscopy and colonoscopy were completed, and no pathology was found toward anemia. We concluded that this mucosal bridge could be a congenital anomaly or that arising from a prior unrecognized (and healed) HIV-related opportunistic infection(s). Noninflammatory esophageal mucosal bridges are very rare. To our knowledge, this is the third case of a noninflammatory esophageal mucosal bridge being reported. The patient is followed-up by hematology to diagnose the etiology and treat anemia.

## Discussion

Despite the first esophageal mucosal bridge being described in 1969, there is still a dearth of epidemiologic data available in the literature.^4^ This, for the most part, is attributed to their rare occurrence. Subsidiary reasons for their uncommon incidence may be due to the fact that many cases present asymptomatically, as was the case with our patient. Given the new, stricter guidelines regarding upper endoscopy, if a patient does not present with alarm signs, a mucosal bridge may go undiagnosed for quite some time.^5^

The pathogenesis of mucosal bridges is also a subject of contention. Current theories postulate an underlying inflammatory process as the main etiology behind its development.^6^ When the walls of inflamed esophagus come into contact with each other, the underlying granulation tissue of each wall develop adhesions, leading to the formation of mucosal bridges.^7^ The well-established relationship of inflammatory bowel disease and mucosal bridges support this theory.^8^ Another theory proposes ulceration as the root cause. The rationale behind this theory is healing of an ulcer by reepithelialization of the mucosal undersurface and subsequent formation of a mucosal tube attached at each end to nonulcerated bowel wall.^9^ In addition to inflammatory bowel disease, other documented associations of esophageal mucosal bridges include lupus, tuberculosis, esophageal candidiasis, reflux esophagitis, and radiation esophagitis.^1,10-13^ Cases have also been found to arise following esophageal variceal sclerotherapy and traumatic insertion of a nasogastric tube.^14,15^ Our patient presented with a noninflammatory mucosal bridge with none of the aforementioned associations. These cases are exceedingly rare, with only 2 other reported cases in the literature to our knowledge. A congenital malformation is considered to be the culprit in these cases.

Symptoms of esophageal mucosal bridges include chest pain, obstructive symptoms, weight loss, and intermittent bleeding.^16^ Currently, there are a variety of treatment options available for eradication of esophageal mucosal bridges. Argon plasma coagulation therapy has been used successfully in several cases.^1,17^ Another technique that has proven effective consists of placing a hemostatic clip at each end of the bridge and incising the center of the bridge with electrocautery.^16,18^ There is no data that support one method over the other, leaving the choice to the discretion of the endoscopist.

## Conclusion

In conclusion, esophageal mucosal bridges are rare entities that can be acquired, or less commonly, developed congenitally. Due to their rarity, there is a lack of epidemiologic data available in the literature. What is clear however is that treatment with argon plasma coagulation or electrocautery has shown to be effective with no reported recurrences. In asymptomatic patients the decision to treat should be deferred to the patient.
